# Improving the Accuracy of Low-Cost Sensor Measurements for Freezer Automation

**DOI:** 10.3390/s20216389

**Published:** 2020-11-09

**Authors:** Kyriakos Koritsoglou, Vasileios Christou, Georgios Ntritsos, Georgios Tsoumanis, Markos G. Tsipouras, Nikolaos Giannakeas, Alexandros T. Tzallas

**Affiliations:** 1 Department of Informatics and Telecommunications, University of Ioannina, GR-47100 Arta, Greece; kkoritsoglou@iti.gr (K.K.); bchristou1@gmail.com (V.C.); gntritsos@uoi.gr (G.N.); gtsoum@uoi.gr (G.T.); giannakeas@uoi.gr (N.G.); 2 Q Base R&D, Science & Technology Park of Epirus, University of Ioannina Campus, GR45110 Ioannina, Greece; 3 Department of Hygiene and Epidemiology, University of Ioannina Medical School, GR-45110 Ioannina, Greece; 4 Department of Electrical and Computer Engineering, University of Western Macedonia, GR-50100 Kozani, Greece; mtsipouras@uowm.gr

**Keywords:** temperature sensor, simple linear regression, temperature monitoring

## Abstract

In this work, a regression method is implemented on a low-cost digital temperature sensor to improve the sensor’s accuracy; thus, following the EN12830 European standard. This standard defines that the maximum acceptable error regarding temperature monitoring devices should not exceed 1 °C for the refrigeration and freezer areas. The purpose of the proposed method is to improve the accuracy of a low-cost digital temperature sensor by correcting its nonlinear response using simple linear regression (SLR). In the experimental part of this study, the proposed method’s outcome (in a custom created dataset containing values taken from a refrigerator) is compared against the values taken from a sensor complying with the EN12830 standard. The experimental results confirmed that the proposed method reduced the mean absolute error (MAE) by 82% for the refrigeration area and 69% for the freezer area—resulting in the accuracy improvement of the low-cost digital temperature sensor. Moreover, it managed to achieve a lower generalization error on the test set when compared to three other machine learning algorithms (SVM, B-ELM, and OS-ELM).

## 1. Introduction

The most critical risk factor for food quality and safety during the cold chain stages is the temperature deviation from the maximum allowed limits. Therefore, it is necessary to continuously monitor and record the temperature, starting from the production process and continuing to the distribution until the end consumer. The term cold chain refers to the equipment, procedures, and information management used for food protection.

According to article 5 of the European Commission regulation 852/2004 [1], all health interest companies must keep a temperature file for documenting the temperature in all stages of their operation. According to article 2 of the European Commission regulation 37/2005 [2], all-temperature measurement devices must fully comply with the EN12830 standard, which sets two basic specifications for temperature sensors. The first specification defines the maximum acceptable measurement error, i.e., the maximum deviation of ±1.0 °C from the actual value. The second specification assumes that the area range for each sensor in which it can take measurements with an acceptable variation is greater or equal to 50 °C.

The experimental part of this work is based on the DS18B20 temperature sensor. This sensor is widely popular, due to its low cost, support of the 1-wire protocol, and good precision over a wide range of temperatures (ranging from −55 °C to 125 °C). The 1-wire serial protocol provides a convenient way to add multiple sensors in a single data line where the master device activates and controls the communication with one or more slave devices [3]. According to the manufacturer’s specifications, the measurement error is determined to be within ±0.5 °C in the range of −10 °C to +85 °C, which increases to ±2.0 °C for the rest of the temperature range [4]. The DS18B20 digital sensors are prevalent, due to their low cost and their ability to communicate via the 1-wire communication protocol. These two characteristics created the conditions for the design and implementation of significantly more economical temperature monitoring and recording systems. Each microcontroller can connect with multiple sensors and simultaneously monitor an equal number of points [4].

In combination with their wide operation range, the above factors have contributed to their rapid implementation of many applications. A significant drawback lies in their usage in temperature monitoring and recording systems intended for the European common market (ECM), due to legal and regulatory requirements restrictions. According to the current regulatory framework, the temperature monitoring devices for refrigerators and freezers must comply with the following EN12830 standard conditions:

- The refrigeration area must operate between 1 °C and 7 °C
- The freezer must operate between −22 °C and −10 °C [2].

Based on the manufacturer’s specifications, the DS18B20 sensor does not satisfy the EN12830 standard requirement, since the measurement uncertainty between −22 °C and −10 °C increases from ±0.5 °C to ±2.0 °C.

In this work, the proposed technique is based on applying *Simple Linear Regression on a DS18B20* (SLR-DS18B20) sensor system, with the goal to improve the DS18B20 digital temperature sensor’s accuracy. SLR-DS18B20 can correct the nonlinear response of the DS18B20 digital temperature sensor using a regression for temperatures below −10 °C to satisfy the EN12830 standard requirements. The proposed technique will develop the systems for temperature monitoring and recording based on the DS18B20 temperature sensor for commercial use inside the European Union (EU).

Several existing methods use linear regression (LR) to calibrate the DS18B20 sensors. Agrimson et al. [5] developed a calibration protocol for the DS18B20 digital temperature sensors used during thermal wake boom experiments. This method utilized a two-point technique to correct each sensor for slope and offset errors. It also used a national institute of standards and technology (NIST) certified thermocouple as standard for the temperature. Hafiz et al. [6] created a control system for receiving real-time height and temperature data regarding crude palm oil (CPO) inside a storage tank. Koestoer et al. [7] proposed a calibration method for the DS18B20 temperature sensor based on Arduino Uno and linear regression. Finally, Chamberlin [8] also used linear regression to calibrate the temperature measurement network based on DS18B20 sensors. Although the above methods use linear regression to calibrate the DS18B20 sensors, they do not conform with the EN12830 standard requirements. The above calibration techniques utilize different temperature ranges than the −22 °C to −10 °C range of the EN12830 standard. This study aims to correct the nonlinear response using linear regression for temperatures below −10 °C to satisfy the EN12830 standard requirements.

A significant number of existing works are trying to improve fiber-optics sensors (which include temperature monitoring amongst their measured data) using various methods. The algorithm proposed by Sun et al. [9] approaches the temperature accuracy problem caused by the Rayleigh noise in optic fiber Raman distributed temperature sensor systems. Lee et al. [10] proposed Gaussian line fitting for improving the accuracy of spectrally distorted Bragg grating sensors. He et al. [11] utilized a signal processing method for reducing the measurement error in a Brillouin distributed optical fiber sensing system. Lalam et al. [12] used a signal-to-noise ratio enhancement in a Brillouin optical time-domain reflectometry system. Soto et al. [13] proposed optical pulse coding as a signal-to-noise ratio improvement, allowing the acquisition of accurate Brillouin intensity and frequency shift measurements at low peak power levels. This is a cost-effective method that avoids high peak power levels utilization. Park and Song [14] designed a fiber Bragg grating (FBG) sensor system having 15 FBGs in its sensor array for distributed temperature monitoring in electrical power systems. In this system, the calculation of the FBG wavelengths took place using interpolation in the temporal Fabry-Perot ITU filter (FPIF) peaks, with the purpose of minimizing the effect of nonlinear wavelength scanning. Loranger et al. [15] utilized the frequency domain Rayleigh scatter to improve the signal strength and the sensitivity in distributed temperature and strain sensing (DTSS). Jin et al. [16] used a calibration method in a distributed optical fiber Raman temperature sensor system for dealing with the measurement error caused by detector nonlinearities. Yan et al. [17] proposed a tunnel fire detection method that utilizes an optical dynamic difference compensation algorithm and visual localization technology for increasing the temperature measurement accuracy. Laarossi et al. [18] studied a high temperature distributed optical fiber sensor based on Raman optical time domain with optical fibers having different coatings. This study aimed to determine the most suitable optical fiber for use in an industrial environment with high temperatures according to distributed temperature sensor measurements. Pan et al. [19] proposed an ensemble empirical mode decomposition denoising algorithm for Raman-based distributed temperature sensors. This approach can enhance the signal to noise ratio without changing spatial resolution. The above methods are focused on improving the accuracy of fiber optic sensors and cannot apply to non-fiber optic-based sensors like the DS18B20 temperature sensor.

Other sensor types improvements include the work from Marinov et al. [20], which investigated the possibilities of improving the accuracy of non-dispersive infrared (NDIR) sensors used in ventilation systems for CO_2_ detection. This research aimed to examine and evaluate the impact of these approaches regarding the accuracy improvement of NDIR CO_2_ sensors. This investigation focused on NDIR sensors without taking into consideration non-NDIR sensors.

A significant number of researchers designed new and novel temperature sensor types. Tang et al. [21] proposed a smart complementary metal-oxide-semiconductor (CMOS) temperature sensor having a digital-assisted readout solution. The digital-assisted readout solution introduces the advantages of increasing the circuit’s compatibility, while achieving high accuracy. Amador et al. [22] utilized a compensation circuit for the design of a Celsius temperature sensor with a purpose to increase its accuracy. Chen et al. [23] used an all-digital linearity enhancement for improving the accuracy of the CMOS smart temperature sensor. Finally, Chen et al. [24] presented a CMOS time-domain smart temperature sensor having one homogeneous delay line and curvature compensation to increase its accuracy. Tan et al. [25] designed and implemented an entirely passive radio-frequency identification (RFID) wireless sensor. This sensor utilizes a power-efficient CMOS circuitry and can achieve on-chip temperature sensing using time-domain signal processing. Heidari et al. [26] proposed a CMOS-based bipolar junction transistor (BJT), optimized for noise-power performance. Wang et al. [27] designed a CMOS-based temperature sensor having thermistor linear calibration and equipped with a circuit for automatic temperature range selection. Shan et al. [28] developed a CMOS-based temperature sensor frontend for thermal monitoring equipped with a dual-slope analog to digital converter (DSADC). Pan et al. [29] designed a high-resolution CMOS temperature sensor for MEMS/quartz frequency references’ temperature compensation. Saffari et al. [30] designed a radio frequency (RF) powered temperature sensor having a non-intermittent operation. Cao et al. [31] designed a physical unclonable function (PUF) based temperature sensor for secure remote temperature sensing environments. Li et al. [32] designed a microprocessor-based system-on-chip (SoC) capable of transforming static random-access memory (SRAM) in the instruction cache to an ambient temperature sensor and a PUF. Although the above sensors provide accurate temperature measurements, they are not low-cost solutions, since they are new sensor types and are not improving the accuracy of already existing low-cost commercial sensors.

The paper structure contains seven main sections (Introduction, Simple Linear Regression, The SLR-DS18B20 Sensor, Experimental Procedure, Simulation Results, Discussion, and Conclusion). The Introduction section contains the description and motivation behind the problem of increasing the DS18B20 temperature sensor’s accuracy to conform with the EN12830 standard, followed by a literature review of other existing methods. The Simple Linear Regression section contains a detailed description of the simple linear regression (SLR) algorithm used to calibrate the DS18B20 temperature sensor. The SLR-DS18B20 sensor architecture section contains a thorough analysis of the proposed system. The Experimental Procedure section describes the process of obtaining the temperature measurements and the parameters that affect measurement accuracy. The Simulation Results section compares the proposed method with a sensor complying with the EN12830 standard. Finally, the last two sections contain the discussion and conclusion of the proposed system.

## 2. Simple Linear Regression

SLR describes the relationship between a dependent variable and an independent variable using a linear model [33]. A simple linear regression model contains one independent variable $X_{i}$ for i=1,…,n subjects. This variable has a linear relationship to the dependent variable $Y_{i}$ and the regression parameters, as seen in the following formula [34]:

(1) $$Y_{i}=aX_{i}+b+e_{i}$$

In this equation, b denotes the *y*-axis intercept, a is the slope, and $e_{i}$ is a random error term. The error term $e_{i}$ is expected to be uncorrelated. The latter’s mean is expected to equal 0, while having a constant variance. Additionally, the SLR algorithm’s efficiency increases when the errors follow the normal distribution [34].

The least-squares method is one of the most commonly used methods for calculating the parameters (i.e., intercept and slope), which define the line that fits the data and can predict Y according to X. Its goal is to find the proper regression parameters which minimize the sum of squared residuals, where value Y is predicted according to the following linear equation:

(2) Y=aX+b

In this equation, X is the independent variable, b denotes the intercept, and a is the slope [34,35,36]. The slope a is calculated according to Equation (3)

(3) $$a=-b\bar{X}+\bar{Y}$$

while the intercept is calculated according to Equation (4).

(4) $$b=\frac{\sum_{i=1}^{n}\left(X_{i}-\bar{X}\right)\left(Y_{i}-\bar{Y}\right)}{\sum_{i=1}^{n}{\left(X_{i}-\bar{X}\right)}^{2}}$$

The mathematical proof for the calculation of a and b parameters’ values can be seen below. It is based in the following sum of squares error formula where $\hat{Y_{i}}$ is the actual value.

(5) $$e=\overset{n}{\underset{i=1}{\sum }}{\left(Y_{i}-\hat{Y_{i}}\right)}^{2}$$

Using Equation (5), the following calculation can be done.

$$\begin{array}{ll} e=\overset{n}{\underset{i=1}{\sum }}{\left(Y_{i}-\hat{Y_{i}}\right)}^{2} & =\overset{n}{\underset{i=1}{\sum }}{\left(Y_{i}-a-bX_{i}\right)}^{2}=\overset{n}{\underset{i=1}{\sum }}\left[\left(Y_{i}+\bar{Y}-\bar{Y}\right)-a-b(X_{i}+\bar{X}-\bar{X}\right){]}^{2}\\ & =\overset{n}{\underset{i=1}{\sum }}{\left[\left(\bar{Y}-a-b\bar{X}\right)+Y_{i}-\bar{Y}-bX_{i}+b\bar{X}\right]}^{2}\\ & =\overset{n}{\underset{i=1}{\sum }}{\left[\left(\bar{Y}-a-b\bar{X}\right)-\left(bX_{i}-b\bar{X}-Y_{i}+\bar{Y}\right)\right]}^{2}\\ & =\overset{n}{\underset{i=1}{\sum }}\left[{\left(\bar{Y}-a-b\bar{X}\right)}^{2}\right]+\overset{n}{\underset{i=1}{\sum }}{\left[\left(bX_{i}-b\bar{X}-Y_{i}+\bar{Y}\right)\right]}^{2}\\ & =n{\left(\bar{Y}-a-b\bar{X}\right)}^{2}+\overset{n}{\underset{i=1}{\sum }}{\left[\left(bX_{i}-b\bar{X}-Y_{i}+\bar{Y}\right)\right]}^{2}\\ & =n{\left(\bar{Y}-a-b\bar{X}\right)}^{2}+\overset{n}{\underset{i=1}{\sum }}{\left[b(X_{i}-\bar{X})-(Y_{i}-\bar{Y})\right]}^{2}\\ & =n{\left(\bar{Y}-a-b\bar{X}\right)}^{2}\\ & +\overset{n}{\underset{i=1}{\sum }}\left[b^{2}{\left(X_{i}-\bar{X}\right)}^{2}-2b\left(X_{i}-\bar{X}\right)\left(Y_{i}-\bar{Y}\right)+{\left(Y_{i}-\bar{Y}\right)}^{2}\right]\\ & =n{\left(\bar{Y}-a-b\bar{X}\right)}^{2}+b^{2}\overset{n}{\underset{i=1}{\sum }}{\left(X_{i}-\bar{X}\right)}^{2}-2b\overset{n}{\underset{i=1}{\sum }}\left(X_{i}-\bar{X}\right)\left(Y_{i}-\bar{Y}\right)\\ & +\overset{n}{\underset{i=1}{\sum }}{\left(Y_{i}-\bar{Y}\right)}^{2}=n{\left(\bar{Y}-a-b\bar{X}\right)}^{2}\\ & +(b^{2}-\frac{2b\sum_{i=1}^{n}\left(X_{i}-\bar{X}\right)\left(Y_{i}-\bar{Y}\right)}{\sum_{i=1}^{n}{\left(X_{i}-\bar{X}\right)}^{2}}+\frac{\sum_{i=1}^{n}{\left(Y_{i}-\bar{Y}\right)}^{2}}{\sum_{i=1}^{n}{\left(X_{i}-\bar{X}\right)}^{2}})\overset{n}{\underset{i=1}{\sum }}{\left(X_{i}-\bar{X}\right)}^{2}\\ & =n{\left(\bar{Y}-a-b\bar{X}\right)}^{2}\\ & +(b^{2}-\frac{2b\sum_{i=1}^{n}\left(X_{i}-\bar{X}\right)\left(Y_{i}-\bar{Y}\right)}{\sum_{i=1}^{n}{\left(X_{i}-\bar{X}\right)}^{2}}+{\left[\frac{\sum_{i=1}^{n}\left(X_{i}-\bar{X}\right)\left(Y_{i}-\bar{Y}\right)}{\sum_{i=1}^{n}{\left(X_{i}-\bar{X}\right)}^{2}}\right]}^{2}\\ & +\frac{\sum_{i=1}^{n}{\left(Y_{i}-\bar{Y}\right)}^{2}}{\sum_{i=1}^{n}{\left(X_{i}-\bar{X}\right)}^{2}}-{\left[\frac{\sum_{i=1}^{n}\left(X_{i}-\bar{X}\right)\left(Y_{i}-\bar{Y}\right)}{\sum_{i=1}^{n}{\left(X_{i}-\bar{X}\right)}^{2}}\right]}^{2})\overset{n}{\underset{i=1}{\sum }}{\left(X_{i}-\bar{X}\right)}^{2}\\ & =n{\left(\bar{Y}-a-b\bar{X}\right)}^{2}+{\left(b-\frac{\sum_{i=1}^{n}\left(X_{i}-\bar{X}\right)\left(Y_{i}-\bar{Y}\right)}{\sum_{i=1}^{n}{\left(X_{i}-\bar{X}\right)}^{2}}\right)}^{2}\\ & +\overset{n}{\underset{i=1}{\sum }}{\left(Y_{i}-\bar{Y}\right)}^{2}\left(1-{\left[\frac{\sum_{i=1}^{n}\left(X_{i}-\bar{X}\right)\left(Y_{i}-\bar{Y}\right)}{\sqrt{\sum_{i=1}^{n}{\left(X_{i}-\bar{X}\right)}^{2}\sum_{i=1}^{n}{\left(Y_{i}-\bar{Y}\right)}^{2}}}\right]}^{2}\right). \end{array}$$

The overbar denotes the average, while n denotes the total number of data. The sum of squares error has been split into a sum of three terms that will be minimized. It can be seen that the first term contains both parameters, while the second term contains parameter b. Moreover, both terms are squared, and they cannot have a value lower than zero, while the third term contains a function of the data and not any parameters. In order to minimize the equation $e=n{\left(\bar{Y}-a-b\bar{X}\right)}^{2}+{\left(b-\frac{\sum_{i=1}^{n}\left(X_{i}-\bar{X}\right)\left(Y_{i}-\bar{Y}\right)}{\sum_{i=1}^{n}{\left(X_{i}-\bar{X}\right)}^{2}}\right)}^{2}+\overset{n}{\underset{i=1}{\sum }}{\left(Y_{i}-\bar{Y}\right)}^{2}\left(1-{\left[\frac{\sum_{i=1}^{n}\left(X_{i}-\bar{X}\right)\left(Y_{i}-\bar{Y}\right)}{\sqrt{\sum_{i=1}^{n}{\left(X_{i}-\bar{X}\right)}^{2}\sum_{i=1}^{n}{\left(Y_{i}-\bar{Y}\right)}^{2}}}\right]}^{2}\right),$ the first two terms must be set to zero. The first term takes the form: $n{\left(\bar{Y}-a-b\bar{X}\right)}^{2}=0\Rightarrow \;\bar{Y}-a-b\bar{X}=0\Rightarrow a=-b\bar{X}+\bar{Y}$ and defines the slope as seen in formula (3). The second term takes the form: ${\left(b-\frac{\sum_{i=1}^{n}\left(X_{i}-\bar{X}\right)\left(Y_{i}-\bar{Y}\right)}{\sum_{i=1}^{n}{\left(X_{i}-\bar{X}\right)}^{2}}\right)}^{2}=0\;\Rightarrow b-\frac{\sum_{i=1}^{n}\left(X_{i}-\bar{X}\right)\left(Y_{i}-\bar{Y}\right)}{\sum_{i=1}^{n}{\left(X_{i}-\bar{X}\right)}^{2}}=\;$0$\;\Rightarrow b=\frac{\sum_{i=1}^{n}\left(X_{i}-\bar{X}\right)\left(Y_{i}-\bar{Y}\right)}{\sum_{i=1}^{n}{\left(X_{i}-\bar{X}\right)}^{2}}$ and defines the intercept b, as seen in Formula (4) [34,35,36,37].

## 3. The SLR-DS18B20 System Architecture

**Figure 1:**
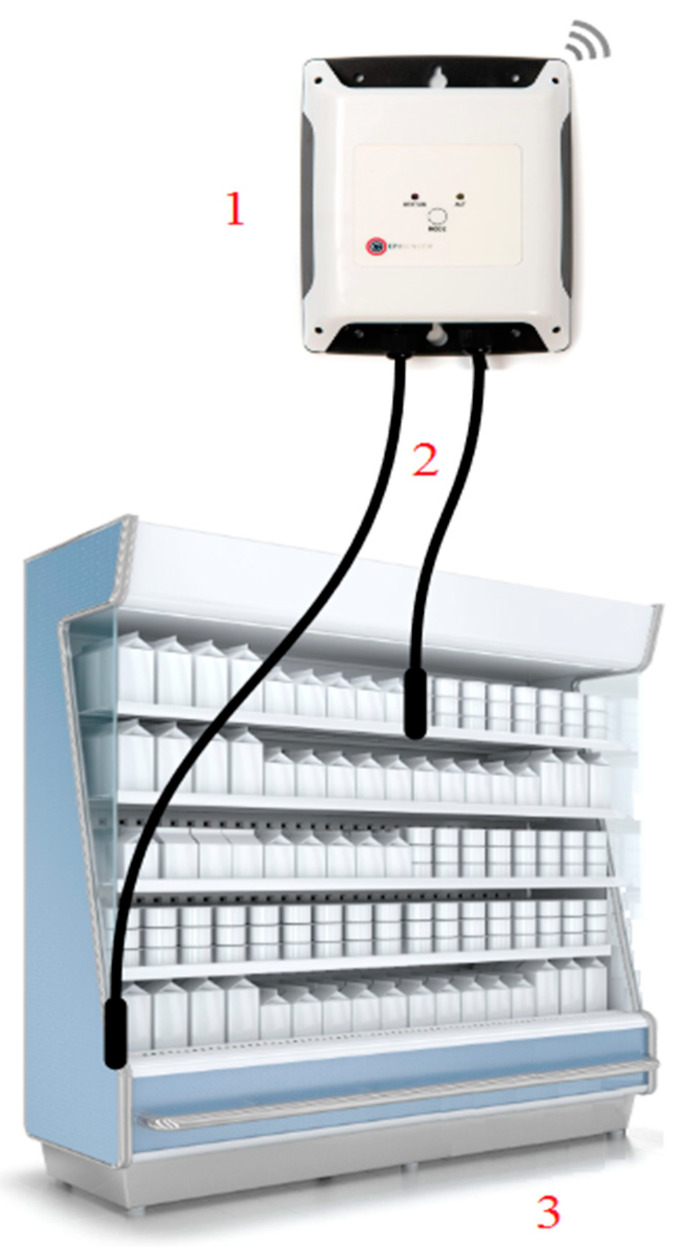
The SLR-DS18B20 System Architecture. This diagram depicts the proposed system’s architecture. The Raspberry Pi Zero W (Device **1**) is connected to the series of DS18B20 sensors (the sensors are given the number **2**) using pins 7, 17, and 25. Pin 7 is responsible for the communication, pin 17 provides 3.3 voltage to the device, while pin 25 is responsible for grounding the circuit. Then, these sensors are inserted into a commercial fridge (device **3**).

The proposed SLR-DS18B20 sensor system utilizes the Raspberry Pi Zero W microcontroller, which connects to a series of DS18B20 sensors using the 1-wire communication protocol. Figure 1 shows the system’s architecture.

The system connects a series of DS18B20 sensors to the Raspberry Pi Zero W microcontroller exploiting 3 of the microcontroller’s pins. The Raspbian operating system that operates on the Raspberry Pi Zero W is implemented with a 1-wire protocol driver for the microcontroller to be able to communicate with the other peripheral devices. The communication is achieved through pin 7 of the microcontroller, while pin 17 provides the device with the appropriate 3.3 voltage, and pin 25 grounds the circuit [38,39].

The reason that led to this choice, compared to other low-cost microcontrollers (Arduino, Esp8266, Esp32, etc.), is that these simple, low-power computers can only run one program at a time. These alternative microcontrollers are designed with ease-of-use in mind, and they are widely utilized in various automation tasks. In addition, Raspberry Pi Zero W is a general-purpose, Linux-based computer system that can run multiple programs simultaneously; thus, providing more features than conventional alternative microcontrollers. Conventional microcontrollers can communicate with personal computers (PCs) via the universal serial bus (USB) port to provide the user with access to their available features, but their use is limited to simple repetitive tasks (e.g., reading the value of various sensors) [38,39]. To be more precise, in cases where multi-tasking or more complex calculations are needed, the utilization of a microcontroller equipped with an operating system with the ability to distribute available resources is necessary. As a result, a more advanced microcontroller like the Raspberry Pi Zero W is the ideal solution for embedded systems or projects requiring more interactivity and processing power. Note that having the abilities of a fully functional computer in a small size offers features that do not exist in conventional microcontrollers [38,39].

Due to a large amount of output data from sampling, available storage space is required. As an indication of the data amount that needs to be stored, note that the experimental part required collecting 650 temperature values from 10 DS18B20 sensors for sequential experiments. The Raspberry Pi Zero W holds a secure digital (SD) memory card and can store all the output data. On the other hand, alternative conventional microcontrollers can maintain only one measurement each time with its value replaced from the next measurement [38,39]

The Raspberry Pi Zero W has a built-in wireless network adapter and can provide network capabilities. This feature is significant for the current project because it allows for data exchange between Raspberry Pi Zero W and the computer, which will perform the statistical processing of the measured values [38,39].

The absence of keyboard, mouse, and screen support does not allow for user interaction with conventional microcontrollers’ applications. Moreover, Raspberry Pi Zero W supports connection with keyboard and mouse via USB or wireless Bluetooth communication protocol. It also holds a graphics processor unit (GPU) equipped with a high-definition multimedia interface (HDMI) output, providing the ability to connect to a screen. All of the above features offer significantly increased user interaction [38,39]. The Raspberry Pi Zero W costs 10$ and is 5$ more expensive than Raspberry Pi Zero, but it offers built-in wireless connectivity that provides ease-of-use for transferring the data between the device and a PC. Moreover, the cheapest version would require the purchase of an adapter for transferring the data, which would exceed the purchase cost of the Raspberry Pi Zero W. A tradeoff regarding the selected microcontroller is its size (65.0 mm × 31.0 mm), which is slightly larger than alternative microcontrollers (e.g., Arduino Nano with size 45 mm × 18 mm [40]) [41].

Conventional microprocessors cannot download the current time and date as they do not have a complete real-time clock (RTC) chip. The absence of RTC makes them unsuitable for this project, since they must record each sample’s time and date. One significant limitation of the Raspberry Pi Zero W is that all software implementation must be done in a Linux environment, which requires substantial knowledge of this operating system.

## 4. Sensors Calibration Procedure

This section describes the process of obtaining the temperature measurements and the parameters that affect measurement accuracy. In order to receive accurate measurements from the proposed system, the sensors must be initially calibrated. A basic calibration principle compares the temperature values obtained from the sensors with some known reference values. In order to conform to this principle, a calibrated sensor that receives the reference values is used. Then, these values are compared to the values measured by the sensors that are going to be calibrated [42]. Accredited laboratories use calibration baths with the purpose to achieve the desired solution temperature for the calibration of the temperature sensors [43]. These devices can heat or cool a solution until it reaches the desired temperature and keeps it until completing the sampling procedure. In the absence of a calibration bath, the solution must achieve the desired temperature under different methods (freezing, cooling, boiling, etc.). The disadvantage of using the aforementioned alternative methods lies in the rapid temperature change of the solution, due to heat exchange with the environment through the receptacle walls containing the solution. Therefore, in order to consider the calibration process reliable, the solution must have the proper heat capacity when its temperature is equal to one of the predefined calibrators. Heat capacity is defined as the heat amount that must be provided to a given material mass to cause a temperature change equal to one temperature measurement unit [44]. In addition, it must remain constant for the time required to receive the values from both the reference sensor and the set of sensors to be calibrated.

**Figure 2:**
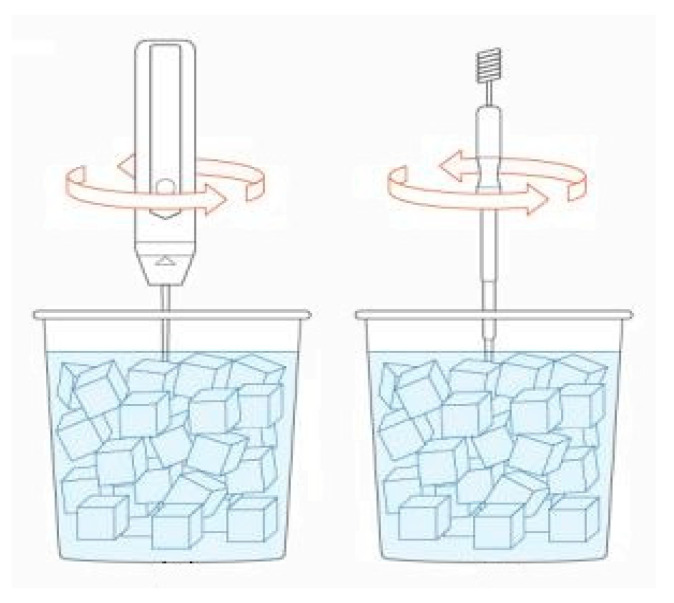
Submerge procedure of the reference sensor and the sensor that is going to be calibrated. Initially, we gently stir and submerge the reference sensor, as seen in the left image. Then, we stir and submerge the sensor that is going to be calibrated (right image).

An important parameter to take into consideration is the depth of sunk sensors [42]. The reference sensor and the other sensors must be at the same depth as the solution’s temperature is not equal for all points. This phenomenon is caused due to heat exchange with the surrounding area. The most common practice is to gently stir the solution to distribute its temperature evenly and then submerge all sensors in the center of the container, as seen in Figure 2.

The difference between the measurement of the device that is about to be calibrated and the measurement of the reference sensor, defines the error. Knowing only the measurement error value cannot conclude for the acceptance or not of the measurement. Therefore, the uncertainty for both the reference sensor and the sensors that are going to be calibrated, should also be considered. In order to minimize the random errors of the sensors that are going to be calibrated, the measurements must be repeated under identical conditions. Employing this method results in the calculation of the mean value and the standard deviation of each measurement. Moreover, a significant parameter is the reference sensor’s accuracy, as a high accuracy value significantly reduces its uncertainty. An acceptable measurement must produce an error and uncertainty sum within acceptable limits. For the measurements to comply with the EN12830 standard, the measurement error and uncertainty sum must be less than 1 °C. [45].

## 5. Experimental Measurements and Simulation Results

This section presents the analysis of the values taken from each sensor. The purpose of this analysis is to determine if the application of linear regression results in the reduction of the relative error, which will make the DS18B20 sensor comply with the EN12830 standard. The measurement process has been split into two parts, with each part corresponding to the following temperature ranges:

1st part. The refrigeration area. It contains temperatures over −10 °C and below or equal to 30 °C.
2nd part. The freezer area. It contains temperatures between −22 °C and −10 °C.

Ten DS18B20 temperature sensors are employed at each temperature area, along with a single high accuracy reference sensor. The sampling procedure, regarding the DS18B20 sensors, used five samples for each measured temperature. The reference sensor used 50 samples for each measured temperature with the purpose of having the same number of entries for both sensor types. The latter configuration’s goal is to evaluate the repeatability and accuracy of the low-cost sensors. The measurements regarding the first part contained 250 entries, while for the second part, the measurements contained 400 entries, forming a dataset of 650 input vectors in total. The reason for using ten DS18B20 temperature sensors instead of one was because the accuracy of each sensor varies slightly, due to the manufacturing process. It was desirable to consider this variability when the dataset was created with the purpose of making the model as accurate as possible.

The sensor accuracy for each temperature measurement was estimated by calculating the absolute error (AE) before and after applying linear regression in comparison to the actual values taken from the reference sensor. The calculation of the AE took place under Formula (7). There, $Y^{\prime }$ is the value taken from the reference sensor, and Y denotes the value taken from the DS18B20 sensor.

(6) $$AE=\left|Y^{\prime }-Y\right|$$

**Figure 3:**
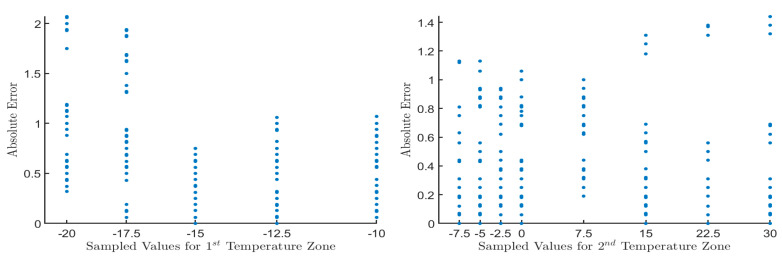
The AE regarding sampled values for both temperature areas. The AE for each sampled value is depicted with blue dots. The *x*-axis shows the temperatures, while on the *y*-axis, the AE calculated using Formula (7). The left diagram shows the 1st temperature zone’s measurement results where the AE ranged from 0 °C to 2.07 °C. The right graph shows the 2nd temperature zone’s measurement results, where the AE went from 0 °C to 2.07 °C.

The AE regarding the sampled values taken from the DS18B20 sensor for both temperature zones before applying linear regression is shown in Figure 3. It is observed there that in the first temperature area, the AE ranged from 0 °C to 2.07 °C, while for the second temperature area, the AE ranged from 0 °C to 1.44 °C.

**Figure 4:**
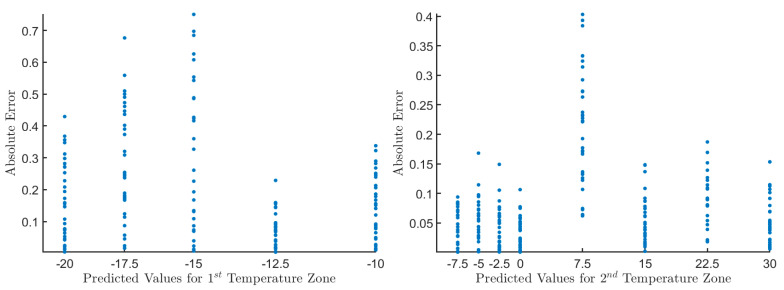
The AE regarding predicted values for both temperature areas. The AE for each sampled value is depicted with blue dots. The *x*-axis shows the temperatures, while on the *y*-axis, the AE, which was calculated using Formula (7). The left diagram shows the 1st temperature zone’s measurement results where the AE ranged from 0 °C to 0.75 °C. The right diagram shows the 2nd temperature zone’s measurement results, where the AE went from 0 °C to 0.4 °C.

The AE regarding the predicted values taken from the DS18B20 sensor for both temperature zones after applying linear regression is shown in Figure 4. It can be seen in Figure 4 that in the first temperature area, the AE ranged from 0 °C to 0.75 °C, while for the second temperature area, the AE ranged from 0 °C to 0.4 °C. This outcome experimentally verifies that linear regression managed to improve the sensor’s accuracy and now can fully comply with the EN12830 standard.

The information in Table 1 shows the mean absolute error (MAE) regarding the sampled and predicted measurements for both temperature zones. The MAE was calculated using Formula (7), where n is the number of entries, Y is the actual value, and $Y^{\prime }$ is the predicted value.

(7) $$MAE=\frac{\sum_{i=1}^{n}\left|Y^{\prime }-Y\right|}{n}$$

This table shows that the linear regression method reduced the MAE by 69% in the first temperature zone and 82% in the second temperature zone. The reduction of the MAE enables the DS18B20 sensor to conform to the EN12830 standard.

The next step is to perform a statistical test to verify if the above error reductions are statistically significant. The statistical test involved creating 95% confidence intervals (Cis) before and after applying linear regression. Then, these Cis were checked for overlapping. The case where no overlaps between the two Cis exists can be understood as strong evidence that the results after applying linear regression are statistically significant [46]. The equation which calculated the 95% CI could be seen below.

(8) $$CI_{95\%}=\;\bar{x}\pm 1.96\frac{s}{\sqrt{n_{smp}}}.$$

In this equation, $\bar{x}$ is the sample mean, s is the standard deviation of the sample, $n_{smp}$ is the sample size, and $\sigma_{M}=\frac{s}{\sqrt{n_{smp}}}$ is the standard error of the mean (SEM). The standard deviation was calculated based on the sampled and predicted coefficient of determination $\left(R^{2}\right)$ values for each temperature zone.

(9) $$R^{2}=1-\frac{\sum_{i=1}^{n}{\left(Y-Y^{\prime }\right)}^{2}}{\sum_{i=1}^{n}{\left(Y-\bar{Y}\right)}^{2}}$$

In this formula, n is the number of entries, Y is the reference value, $Y^{\prime }$ is either the sampled or predicted values, and $\bar{Y}$ is the average of all reference values.

**Figure 5:**
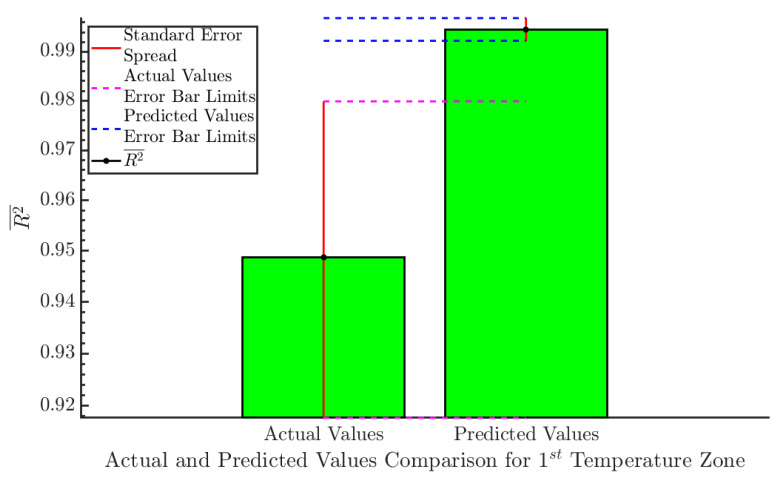
Comparison between actual and predicted values for the 1st temperature zone. This diagram compares the $\bar{R^{2}}$ for the actual and predicted values. The 95% CI for each category can be seen with a red vertical line. Each CI’s limits are depicted with magenta (actual values), and blue (predicted values) dashed lines. The two Cis do not overlap, which is a strong indication that the error reduction achieved after the application of linear regression is statistically significant.

The average $R^{2}$ $\left(\bar{R^{2}}\right)$ for the actual and predicted values of the 1st temperature zone can be seen in Figure 5. The $\bar{R^{2}}$ for the sample values was 0.949, which increased to 0.995 for the predicted values. Both Cis do not overlap, which can be understood as strong evidence that the results after applying linear regression are statistically significant.

**Figure 6:**
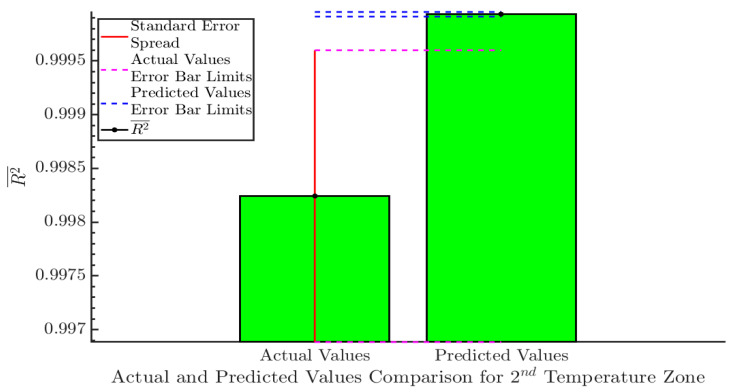
Comparison between actual and predicted values for the 2nd temperature zone. This diagram compares the $\bar{R^{2}}$ for the actual and predicted values. The 95% CI for each category can be seen with a red vertical line. The limits for each CI are depicted with magenta (actual values), and blue (predicted values) dashed lines. The two Cis do not overlap, which is a strong indication that the error reduction achieved after the application of linear regression is statistically significant.

The average $R^{2}$ $\left(\bar{R^{2}}\right)$ for the actual and predicted values of the 2nd temperature zone can be seen in Figure 6. The $\bar{R^{2}}$ for the sample values was 0.99824, which increased to 0.99994 for the predicted values. Both Cis do not overlap, which can be understood as strong evidence that the results after applying linear regression are statistically significant.

## 6. Comparative Study

The SLR has been tested against three existing machine learning techniques. These techniques are support vector machine (SVM) and two variations of the extreme learning machine (ELM) algorithm: Bidirectional extreme learning machine (B-ELM) and on-line sequential extreme learning machine (OS-ELM). SVM is a supervised learning algorithm created in 1963 by Vladimir N. Vapnik and Alexey Ya. Chervonenkis. The original SVM algorithm has been improved, since its creation in 1963, with the current standard incarnation being proposed by Cortes and Vapnik [47]. The SVM algorithm constructs a separating hyperplane or a series of hyperplanes in a high or infinite-dimensional space for use in various tasks, including classification and regression [48]. The ELM algorithm was created by Huang et al. [49] and can train a single layer neural network (SLNN) very fast. Initially, it randomly chooses the hidden layer neurons’ weights and thresholds, and with the help of the Moore-Penrose pseudo-inverse, is able to calculate the output node weights. One characteristic of ELM is that it tends to reach a low training error with a small norm of output weights, which according to Bartlett’s theory [50], leads to better generalization performance [51]. The Bi-directional ELM (B-ELM) proposed by Yang et al. [52] can reduce the number of hidden layer units by incrementally updating some hidden layer nodes during the network growth. OS-ELM proposed by Huang et al. [53] is a modified version of the ELM algorithm, which can work with sequential data.

The experimental part includes comparing the proposed SLR-DS18B20 systems and three machine learning methods (SVM, B-ELM, and OS-ELM). The design of these experiments involved using 10-fold cross-validation to estimate the performance of SLR-DS18B20 on unseen data. The parameters used for each compared method can be seen in Table 2.

The SVM method used linear and radial basis function (RBF) kernel types. B-ELM used sigmoid and sinusoid activation functions, while OS-ELM used sigmoid, sinusoid, and RBF activation functions. The ELM-based methods used random hidden weights and thresholds (w,θ∈[−1, 1]) taken from the uniform distribution, the number of hidden neurons was set to 100, and the experiments were repeated ten times.

As seen in the comparison results of Table 3, the proposed SLR-DS18B20 method was able to achieve a lower mean square error (MSE) than the other compared methods (the lowest MSE value is shown in bold). The calculation of MSE is done according to the following formula.

(10) $$MSE=\frac{1}{kp}\overset{k}{\underset{i=1}{\sum }}\left(\overset{p}{\underset{j=1}{\sum }}{\left(t_{i}^{j}-y_{i}^{j}\right)}^{2}\right)$$

In this formula, k defines the number of folds, p defines the number of validation patterns, $t_{i}^{j}$ defines the current pattern target network output value for the current fold and $y_{i}^{j}$ defines the current pattern network output value for the current fold.

## 7. Discussion

The proposed system can improve the DS18B20 digital temperature sensor’s accuracy to satisfy the EN12830 standard requirements. The accuracy improvement will create temperature monitoring and recording systems based on the low-cost DS18B20 temperature sensor for commercial use inside the EU.

As seen from the experimental results in Section 5, the proposed method managed to get significantly lower MSE than the other compared methods, which, in most cases, was at least one order less. One exception was the SVM method, which produced a higher error than SLR-DS18B20, but it had the same order. The 0.0003505 MSE of the proposed method was derived by averaging the MSE from all input test patterns for each fold. Then, the experiments regarding the ELM-based methods were repeated ten times with different values for the hidden layer weights and thresholds to avoid any bias, due to the randomization of the hidden units. The overall MSE involved averaging the MSE from all experiment runs. One typical value regarding the MSE formula is to set the number of folds to 10 (for relatively large datasets) or 5 (for small datasets).

The experimental procedure involved comparing the low-cost EN12830 temperature sensor with a high accuracy reference temperature sensor before and after applying linear regression. The experimental procedure followed a basic calibration principle where the temperature values obtained from the sensors had been compared with some known reference values. The experimental results in Section 6 showed that the proposed linear regression calibration method could increase the DS18B20 sensor’s accuracy in two temperature zones to comply with the EN12830 standard requirements. The temperature zones are: (i) The refrigeration area, which must operate between 1 °C and 7 °C; and (ii) the freezer area, which must operate between −22 °C and −10 °C. These results were statistically significant since the 95% CIs from the actual and predicted values for both temperature zones did not overlap. In Section 7, a comparative study of the proposed SLR-DS18B20 sensor system with three existing machine learning methods was conducted. The outcome from this comparison experimentally verified that the proposed SLR-DS18B20 system could provide a fast, low-cost solution for improving the accuracy of the DS18B20 temperature sensor because it was able to achieve a lower MSE than the other three alternative methods.

## 8. Conclusions

The proposed SLR-DS18B20 sensor system can reduce the DS18B20 digital temperature sensor’s MAE by 82% for the refrigeration area and 69% for the freezer area using a simple, non-computationally intensive method. This reduction is achieved by correcting its nonlinear response using linear regression to comply with the EN12830 standard. Conforming to this standard makes this sensor or other low-cost sensors eligible for use in freezers sold within the ECM. The proposed system utilized the low-cost Raspberry Pi Zero W microcontroller, which connected with several DS18B20 sensors through the 1-wire communication protocol.

The objective of the proposed calibration method was to improve the accuracy and repeatability of the measuring instruments. After completing the calibration process, each DS18B20 digital temperature sensor had its accuracy significantly improved. The predicted values calculated in the Simulation Results section are shown to be much closer to the real ones compared to the actual values before calibration was applied. Therefore, the DS18B20 digital temperature sensors can be used safely in refrigeration temperature monitoring and recording systems intended for the ECM, which is the primary objective of this work.

## 9. Future Work

Future directions include the hybridization of the proposed method with machine learning methods to achieve even greater accuracy and the implementation of this method to other sensor types (e.g., humidity sensor).

## Figures and Tables

**Table 1:** The comparison between the sampled and predicted measurements for both temperature zones.

	Sampled Measurements	Predicted Measurements
1st Temperature Zone	0.64	0.41
2nd Temperature Zone	0.19	0.007

**Table 2:** Parameter settings.

Method	Parameter Name	Symbol	Values/Types
SVM	Kernel	ker	ker={Linear, RBF}
B-ELM	Output Function	g	$g_{B-ELM}=\left\{Sigmoid,\;Sinusoid\right\}$
OS-ELM	Output Function	g	$g_{OS-ELM}=\left\{Sigmoid,\;Sinusoid,\;RBF\right\}$
B-ELM and OS-ELM	Input Weights Matrix	w	w∈[−1, 1]
	Input Thresholds Matrix	θ	θ∈[−1, 1]
	Hidden Layer Units No	*n*	n∈100
	Experiments No	expNo	expNo=10

**Table 3:** Comparison Results.

Method	MSE
SVM (Linear)	0.0006991
SVM (RBF)	0.0003568
B-ELM (Sigmoid)	0.0020735
B-ELM (Sinusoid)	0.0119922
OS-ELM (Sigmoid)	0.0018569
OS-ELM (Sinusoid)	0.0010747
OS-ELM (RBF)	0.0010636
SLR-DS18B20	0.0003505
